# Risk Factors for Cataracts Treated Surgically in Postmenopausal Women

**DOI:** 10.1016/j.ophtha.2016.04.037

**Published:** 2016-08

**Authors:** Sarah Floud, Hannah Kuper, Gillian K. Reeves, Valerie Beral, Jane Green

**Affiliations:** 1 Cancer Epidemiology Unit, Nuffield Department of Population Health, University of Oxford, Oxford, United Kingdom; 2 International Centre for Evidence in Disability, London School of Hygiene and Tropical Medicine, London, United Kingdom

**Keywords:** BMI, body mass index, CI, confidence interval, HT, hormone therapy, NHS, National Health Service, OPCS-4, Office of Population Censuses and Survey's classification of surgical operations and procedures, fourth revision, RR, relative risk, SD, standard deviation, UV, ultraviolet

## Abstract

**Purpose:**

To identify risk factors for cataracts treated surgically in postmenopausal women.

**Design:**

Population-based, prospective cohort study.

**Participants:**

A total of 1 312 051 postmenopausal women in the UK Million Women Study, aged 56 years on average (standard deviation [SD], 4.8), without previous cataract surgery, hospital admission with cataracts, or cancer at baseline, were followed for cataracts treated surgically.

**Methods:**

Cox regression was used to calculate adjusted relative risks (RRs) for cataract surgery by lifestyle factors, treatment for diabetes, reproductive history, and use of hormonal therapies.

**Main Outcome Measures:**

Cataract surgery identified by linkage to central National Health Service (NHS) records for inpatient and day-patient admissions (Hospital Episode Statistics for England and Scottish Morbidity Records in Scotland).

**Results:**

Overall, 89 343 women underwent cataract surgery during an average of 11 (SD, 3) years of follow-up. Women with diabetes were at greatest risk (diabetes vs. no diabetes RR, 2.90; 95% confidence interval [CI], 2.82–2.97). Other factors associated with an increased risk of cataract surgery were current smoking (current smokers of ≥15 cigarettes/day vs. never smokers RR, 1.26; 95% CI, 1.23–1.30) and obesity (body mass index [BMI] ≥30 vs. <25 kg/m^2^; RR, 1.12; 95% CI, 1.10–1.14).

**Conclusions:**

Diabetes, smoking, and obesity were risk factors for cataract surgery. Alcohol use, physical activity, reproductive history, and use of hormonal therapies had little, if any, association with cataract surgery risk.

Cataract is the second leading cause of partial sight and blindness in the United Kingdom, exceeded only by age-related macular degeneration.^1^ Identifying potential risk factors for cataract is important given that the only treatment for cataract is the surgical removal of the lens. The most important risk factor for cataract formation is age.^2^ Other risk factors that have been identified are diabetes, smoking, ultraviolet (UV) light, and steroid use,^2^, ^3^, ^4^, ^5^, ^6^, ^7^ but there is less evidence on the role of factors such as body mass index (BMI), physical activity, reproductive history, use of hormonal therapies, and alcohol consumption.^4^, ^8^, ^9^, ^10^, ^11^

Cataract surgery is the most common surgical procedure in the National Health Service (NHS) in England, with more than 340 000 operations carried out in 2013 and 2014.^12^ Rates of cataract surgery in England have increased rapidly since 1990 because of the widespread uptake of phacoemulsification and day case surgery.^11^ For people aged more than 60 years, rates are higher for women compared with men.^11^ It has been proposed that this higher rate of surgery results from higher incidence of cataracts in postmenopausal women compared with men of a similar age because of hormonal differences between men and women.^13^, ^14^, ^15^ However, epidemiologic evidence on the relationship between cataracts and reproductive and hormonal factors, including use of hormone therapy (HT) for menopause, is limited and inconsistent.^4^, ^16^, ^17^

In a large cohort of postmenopausal women from the United Kingdom, with virtually complete long-term follow-up for surgical procedures through linkage to hospital records, we have examined potential risk factors for cataracts treated surgically, including treatment for diabetes, lifestyle, and reproductive and hormonal factors.

## Methods

### Data Collection and Definitions

The Million Women Study is a population-based prospective study of women in the United Kingdom. Details of the design and methods of the study have been described.^18^ Briefly, 1.3 million women aged 50 to 64 years were invited for breast cancer screening at NHS clinics in England and Scotland and were recruited to the study between 1996 and 2001 by completing a questionnaire, which included questions on various socioeconomic, lifestyle, reproductive, and hormonal factors. The respondents gave written consent to participate and for follow-up though their NHS medical records; ethical approval was provided by the Oxford and Anglia Multi-Centre Research Ethics Committee. Study questionnaires and details of the study data and access policies can be viewed on the website (www.millionwomenstudy.org).

### Follow-up

Individuals in the study are linked by their unique NHS identification number to NHS Central Registers, through which they are followed for emigration, death, and cancer registration, and to NHS hospital admissions databases: Hospital Episode Statistics for England and Scottish Morbidity Records for Scotland. The databases include information on both inpatient stays and day-case admissions (e.g., for surgical procedures). Follow-up is 99% complete; only 18 970 women (1%) have been lost to follow-up in the entire cohort, and they have been included in analyses up to the date of loss to follow-up. Information on the date and type of procedures associated with each hospital admission is provided, coded to the Office of Population Censuses and Survey's classification of surgical operations and procedures, fourth revision (OPCS-4). Linked data for England are provided to the cohort through the Health and Social Care Information Centre and the Office for National Statistics, and for Scotland by the NHS Information Services Division.

### Statistical Analysis

Analyses were restricted to postmenopausal women. Those who reported at recruitment that they had experienced natural menopause (49%) or who had undergone a bilateral oophorectomy (6%) were defined as postmenopausal and included in follow-up from recruitment. Women who were premenopausal, perimenopausal, or of unknown menopausal status at recruitment were assumed to be postmenopausal after they reached the age of 55 years and were included in follow-up from age 55 years, because 96% of women in this cohort with a known age at natural menopause were postmenopausal by that age.^19^ Women were excluded from the analysis if their linked hospital records showed that they had previous cataract surgery or were admitted to a hospital with cataracts before recruitment, or if the cancer registration records showed that they had preexisting cancer, with the exception of nonmelanoma skin cancer. For these analyses, cases were defined as the first hospital record (day-case or overnight admission) of cataract surgery (OPCS-4:C71–C75) occurring after recruitment into the study. Women were followed until the date of first cataract surgery, date of death, or the end of the hospital admissions follow-up period (March 2011 for England and December 2008 for Scotland).

Cox proportional hazards models were used to estimate relative risks (RRs) and 95% confidence intervals (CIs) for cataracts treated surgically according to area deprivation (quintiles, based on the Townsend index, a score incorporating census area data for employment, car ownership, home ownership, and household overcrowding^20^), educational qualifications (“tertiary” [college or university], “secondary” [A levels or O levels, usually obtained at age 18 and 16 years respectively], “technical” [nursing, teaching, clerical, or commercial], “no qualifications”), smoking status (never, past, current <15 cigarettes per day, current ≥15 cigarettes per day), BMI (<25, 25–29, ≥30 kg/m^2^), alcohol intake (<2, 2–14, ≥15 units per week [unit ∼10 g alcohol]), strenuous physical activity (rarely/never, some), self-reported treatment for diabetes (yes, no), age at menarche (≤12, 13, 14, ≥15 years), parity (nulliparous, parous), number of children (1, 2, 3, ≥4), duration of oral contraceptive use (<5, ≥5 years), and use of HT for menopause (never, ever). All variables were as reported at recruitment. The underlying time variable was attained age, and all analyses were routinely stratified for the recruitment region (10 geographic regions). For RRs reported as multiply adjusted, analyses were mutually adjusted for all other potential risk factors using the categories described (except for parity, which was adjusted for using 2 categories: parous/nulliparous). Missing values of adjustment factors (<6% for all variables) were included as a separate category.

In an additional analysis, we investigated the association between cataracts treated surgically and the 2 main types of HT: estrogen-only or estrogen-progestogen. Women reporting current use were classified according to most recent type reported, and analyses accounted for changes in use during follow-up. Women were initially classified using information provided at recruitment, and those who provided updated information on HT use on the second study questionnaire (on average 3 years after recruitment) were then reclassified using this updated information. For all women, the period of follow-up was censored at 48 months after last report of HT use. All analyses used Stata 14.1 (StataCorp LP, College Station, TX).

## Results

After excluding 4150 women with a hospital record of cataract surgery before recruitment, 166 women with a previous hospital admission diagnosis of cataract, and 44 781 women with preexisting cancer, these analyses included prospective data on cataract surgery in 1 312 051 postmenopausal women. Over a mean follow-up period of 10.7 years (standard deviation [SD], 2.6 years) per woman, 89 343 women (6.8%) underwent cataract surgery, corresponding to an incidence rate of 6.38 (95% CI, 6.33–6.41) per 1000 person-years.

Table 1 shows the characteristics of the study population at baseline. Mean age at recruitment was 56.1 years (SD, 4.8 years); 21% reported that they were current smokers; mean BMI was 26.2 kg/m^2^ (SD, 4.7); 59% reported ever using oral contraceptives; and 34% were currently using HT.

Table 2 shows the RR of cataracts treated surgically by various categories of socioeconomic and lifestyle factors, as well as treatment for diabetes. Overall, 2% (31 612) of the women in the study population reported being treated for diabetes, and this was the strongest risk factor for cataracts treated surgically (multiply adjusted RR for diabetes vs. no diabetes, 2.90, 95% CI, 2.82–2.97). Both past and current smoking at recruitment were associated with increased risk of cataracts treated surgically, with multiply adjusted RRs compared with never smokers of 1.10 (95% CI, 1.08–1.12) in past smokers, 1.12 (95% CI, 1.09–1.14) in current smokers of less than 15 cigarettes per day, and 1.26 (95% CI, 1.23–1.30) in current smokers of 15 or more cigarettes per day. Obesity was associated with a small increase in risk of cataracts treated surgically; this association was stronger in the minimally adjusted analysis (adjusted for age and region only) but remained significant after adjustment for other factors, including treatment for diabetes (multiply adjusted RR, 1.12; 95% CI, 1.10–1.14 for women with BMI ≥30 kg/m^2^ compared with <25 kg/m^2^). In analyses restricted to those not reporting treatment for diabetes, the association was similar (multiply adjusted RR, 1.15; 95% CI, 1.12–1.17 for women with BMI ≥30 kg/m^2^ compared with <25 kg/m^2^).

Women who reported doing some strenuous physical activity every week had a slightly lower risk of cataracts treated surgically compared with women who reported rarely or never engaging in strenuous physical activity (multiply adjusted RR, 0.90; 95% CI, 0.88–0.91). Alcohol consumption of 2 units or more per week was weakly associated after adjustment, with a slightly lower risk of cataracts treated surgically compared with consumption of less than 2 units per week, but there was no suggestion of a trend by amount of alcohol.

In minimally adjusted analyses (adjusted for age and region only) both deprivation and lack of educational qualifications were associated with higher risks of cataracts treated surgically. However, after adjustment for other factors, there was no association with education, and the association with deprivation was attenuated.

Table 3 shows the RR of cataracts treated surgically by reproductive history and use of hormonal therapies. There were no major differences in the risk of cataracts treated surgically according to age at menarche or use of oral contraceptives. Parous women seemed to be slightly less likely to undergo cataract surgery than nulliparous women, but there was no evidence for a trend in risk by number of children among parous women. Ever users of HT at recruitment were slightly more likely than never users to undergo surgery for cataract (multiply adjusted RR, 1.07; 95% CI, 1.06–1.09). In analyses looking at risk in relation to HT type (in 1228856 women; 6444986 total follow-up years), current users of estrogen-only HT had an RR of 1.08 (95% CI, 1.04–1.13) for cataracts treated surgically, and current users of estrogen-progestogen HT had an RR of 1.02 (95% CI, 0.98–1.07).

## Discussion

In this large prospective study of postmenopausal women, previously reported associations between both diabetes and smoking and an increased risk of cataracts were confirmed. There was also evidence of an increased risk for obese women. Physical activity, alcohol intake, reproductive factors, and hormonal factors had little, if any, association with cataracts treated surgically: the RRs were small in magnitude, and the effect of residual confounding cannot be ruled out.

### Diabetes and Lifestyle Factors and Risk of Cataracts Treated Surgically

Previous research has shown that cataracts develop at an earlier age in people with diabetes.^3^, ^21^, ^22^ The pathogenesis of cataracts in diabetic individuals is not fully understood.^3^, ^23^ Lifestyle factors that have been identified as risk factors for cataracts are smoking and UV light exposure, both of which are thought to affect cataract formation through oxidation.^4^, ^5^, ^24^, ^25^, ^26^ In addition, the use of corticosteroids, both systemic and inhaled, has been identified as a risk factor for cataract formation.^2^, ^6^, ^7^ This study did not have information on UV light exposure or use of corticosteroids at baseline, but our results for smoking confirm the higher risks found in other studies.^24^, ^25^, ^26^ Our results also provide further confirmatory evidence that there is an increased risk of cataracts in past smokers as well as in current smokers.^24^

Two recent meta-analyses have examined the relationship between alcohol intake and the risk of cataract or cataracts treated surgically.^27^, ^28^ The first meta-analysis in 2014 of 7 prospective cohort studies did not find alcohol to be a statistically significant risk factor for cataract surgery or cataracts.^28^ The second meta-analysis in 2015 of 5 prospective studies and 5 case-control studies reported a borderline increased risk of cataracts for heavy alcohol consumption (classified as >14 units per week), but confounding by smoking was seen.^27^ We did not find a clearly increased risk of cataracts treated surgically related to heavy alcohol consumption, but there are few heavy alcohol drinkers in this cohort. The slight decreased risk in our study for moderate alcohol consumption is consistent with the previous evidence of a lack of association.^27^, ^28^

We found evidence that obesity (BMI ≥30 kg/m^2^) may weakly increase the risk of cataracts treated surgically, but there was no increased risk related to overweight (25–29 kg/m^2^). This is in line with the results of a meta-analysis of 6 prospective studies that showed that the development of all types of cataract (nuclear, cortical, or posterior subcapsular) was associated with obesity.^29^ It is unclear how much of the association with obesity is due to clinical or subclinical diabetes.^4^, ^8^, ^9^, ^10^, ^29^ However, when women who reported treatment for diabetes were excluded, the association we found between BMI and cataracts treated surgically did not change, which suggests that obesity may be an independent risk factor.

Previous evidence on the potential effect of physical activity is sparse, with only 3 prospective studies having been carried out,^30^, ^31^ and only 1 in the general population.^32^ The evidence from our study of a slightly lower risk associated with doing some strenuous physical activity agrees with previous evidence. However, this lower risk associated with physical activity could be due to reverse causation bias, because women with cataracts might be less likely to participate in strenuous physical activity because of visual impairment.

### Hormonal and Reproductive Factors and Risk of Cataracts Treated Surgically

A recent meta-analysis of 4 prospective cohort studies concluded that use of HT for menopause was associated with a decreased risk of diagnosed cataract for ever users (pooled OR, 0.83; 95% CI, 0.71–0.97 compared with never users).^16^ In contrast, a prospective study from Sweden, with more than 30 000 women and 4324 cataract surgery cases, not included in the meta-analysis, found that HT use was associated with an increase in risk of cataracts treated surgically (RR, 1.14; 95% CI, 1.07–1.21 for ever users compared with never users).^17^ In our multiply adjusted analyses, ever users of HT at recruitment were slightly more likely than never users to undergo surgery for cataract (RR, 1.07; 95% CI, 1.06–1.09), with no material difference in risk between users of estrogen-only and estrogen-progestogen HT. These findings should be interpreted with caution because such small increases (or decreases) in RR may well reflect residual confounding. The present results suggest that a strong relationship between HT use and cataracts is unlikely.

Few other studies have investigated the association of reproductive factors with cataracts, and these have had mixed results.^33^, ^34^, ^35^ We found no evidence to support a role of the reproductive factors studied (age at menarche and parity) in relation to risk of cataracts treated surgically; while a statistically significant reduction in risk was found for parous compared with nulliparous women, there was no evidence for a trend in risk by number of children among parous women, and, again, residual confounding could well explain a small apparent association.

### Study Strengths and Limitations

The strengths of this study are the large sample size and the long average follow-up period of 11 years. This is the largest single study examining risk factors for cataracts treated surgically. With approximately one quarter of eligible women in the United Kingdom participating in the Million Women Study and the power to examine risk factors across a full range of population values, the study can be considered representative of the UK population of middle-aged women. The incidence rates of cataract surgery (6.38 per 1000) are similar to those reported for the whole UK population in 2004^11^ (6.37 per 1000). The inclusion of socioeconomic, lifestyle, reproductive, and hormonal factors, as well as treatment for diabetes, enabled us to adjust for many risk factors at the same time. The use of linked routinely collected health records of cataract surgery as the end point of interest is both a strength—allowing near-complete, unbiased, long-term, cost-effective follow-up in a large cohort—and a limitation, in that hospital records provide limited information on clinical details such as type of cataract and extent of visual loss.

In general, the majority of surgeries in the United Kingdom are performed within the NHS,^36^ but some women in the cohort may have undergone privately funded cataract surgery outside the NHS and therefore would not be identified by the record linkage to NHS data. In 1997 and 1998, an estimated 14% of lens operations in England and Wales were privately funded and carried out in private hospitals.^36^ This estimate predates follow-up for this study, but we are not aware of more recent estimates. Misclassification of cases is unlikely to have had a substantial effect on power to detect risk factor associations, but, together with residual confounding, this could account for the association between deprivation and cataracts treated surgically.

The risk factor associations identified in the current article in relation to surgery for cataracts may not be identical to those related to the development of cataract; although minimally invasive surgery for cataract is widely available in the United Kingdom, comorbidities that may be related to the risk factors studied are likely to affect uptake of surgery. For example, cataract surgery can be performed to assist the management of other eye conditions, such as diabetic retinopathy.^37^ However, our findings for diabetes and smoking are consistent with previous studies of both cataract^21^, ^24^, ^38^ and surgery for cataract.^22^, ^24^, ^25^

In conclusion, cataracts are the second leading cause of partial sight and blindness in the United Kingdom, and cataract surgery is the most common operation performed in the United Kingdom. Therefore, it is important to identify any risk factors that may be modifiable. For the women in this large prospective cohort study in the United Kingdom, diabetes and smoking were associated with a higher risk of undergoing cataract surgery. There was also evidence of a small increase in risk for obese women. Alcohol use, physical activity, reproductive history, and use of hormonal therapies had little, if any, association with cataract surgery risk.

## Figures and Tables

**Table 1:** Baseline Characteristics of Women Who Subsequently Underwent Cataract Surgery and of All Women Included in the Analysis

Characteristics at Baseline	Women Who Underwent Cataract Surgery during Follow-up	All Women
No. of women	89 343	1 312 051
Mean age at recruitment, yrs (SD)	59.5 (5.1)	56.1 (4.8)
Percentage in most deprived quintile (n)	23.3 (20 654)	19.9 (258 798)
Percentage with no qualifications (n)	50.5 (43 657)	43.9 (561 001)
Percentage current smokers (n)	19.4 (16 177)	20.5 (253 411)
Mean BMI, kg/m^2^ (SD)	26.7 (4.9)	26.2 (4.7)
Mean alcohol intake, units/wk (SD)	3.5 (5.0)	4.1 (5.3)
Percentage rarely/never strenuously active (n)	55.2 (46 960)	48.7 (614 944)
Percentage treated for diabetes (n)	7.5 (6671)	2.4 (31 612)
Mean parity (SD)	2.2 (1.4)	2.1 (1.2)
Mean age at menarche, yrs (SD)	13.1 (1.6)	13.0 (1.6)
Percentage ever used oral contraceptives (n)	47.7 (41 916)	59.3 (769 595)
Percentage current user of HT (n)	29.5 (25 934)	33.5 (434 135)

BMI = body mass index; HT = hormone therapy; SD = standard deviation.

**Table 2:** Relative Risk of Cataracts Treated Surgically Associated with Various Socioeconomic Factors, Lifestyle Factors, and Diabetes

	Population at Risk	Cases	RR (95% CI) Minimally Adjusted∗	RR (95% CI) Multiply Adjusted†
Area deprivation				
1: least deprived	262 316	15 388	1.00	1.00
2	261 515	16 751	1.04 (1.02–1.07)	1.03 (1.00–1.05)
3	259 789	17 451	1.10 (1.08–1.13)	1.07 (1.04–1.09)
4	260 093	18 447	1.18 (1.16–1.21)	1.11 (1.09–1.13)
5: most deprived	258 798	20 654	1.38 (1.35–1.41)	1.22 (1.22–1.25)
Educational qualifications				
Tertiary	167 904	9481	1.00	1.00
Secondary	333 445	19 299	1.01 (0.98–1.03)	0.98 (0.96–1.01)
Technical	215 692	13 983	1.04 (1.01–1.07)	0.99 (0.96–1.02)
No qualifications	561 001	43 657	1.17 (1.14–1.19)	1.02 (0.99–1.04)
Smoking				
Never	630 939	42 007	1.00	1.00
Past	350 569	25 211	1.13 (1.11–1.14)	1.10 (1.08–1.12)
Current <15	129 961	8318	1.15 (1.13–1.18)	1.12 (1.09–1.14)
Current 15+	123 450	7859	1.35 (1.32–1.39)	1.26 (1.23–1.30)
BMI				
<25 kg/m^2^	575 282	35 589	1.00	1.00
25–29 kg/m^2^	445 121	30 821	1.06 (1.05–1.08)	1.02 (1.01–1.04)
30+ kg/m^2^	223 065	17 645	1.30 (1.27–1.32)	1.12 (1.10–1.14)
Alcohol (units per week)				
<2	557 045	43 447	1.00	1.00
2–14	679 389	41 331	0.86 (0.85–0.87)	0.93 (0.91–0.94)
15+	65 314	3613	0.87 (0.83–0.89)	0.92 (0.89–0.96)
Strenuous physical activity				
Rarely/never	614 944	46 960	1.00	1.00
Some	647 872	38 177	0.83 (0.82–0.84)	0.90 (0.88–0.91)
Treatment for diabetes				
No	1 279 660	82 589	1.00	1.00
Yes	31 612	6671	3.15 (3.07–3.23)	2.90 (2.82–2.97)

BMI = body mass index; CI = confidence interval; RR = relative risk.

∗ Estimates of RR are adjusted by age and region of residence.

† Estimates of RR are adjusted by age and region of residence plus deprivation, educational attainment, smoking, BMI, alcohol intake, strenuous physical activity, treatment for diabetes, age at menarche, parity, duration of oral contraceptive use, and HT use as appropriate.

**Table 3:** Relative Risk of Cataracts Treated Surgically Associated with Reproductive History and Use of Hormonal Therapies

	Population at Risk	Cases	RR (95% CI) Minimally Adjusted∗	RR (95% CI) Multiply Adjusted†
Age at menarche				
≤12 yrs	489 916	32 190	1.00	
13 yrs	312 596	20 354	0.93 (0.92–0.95)	0.96 (0.95–0.98)
14 yrs	264 093	18 189	0.94 (0.93–0.96)	0.96 (0.94–0.98)
15+ yrs	218 444	16 634	0.99 (0.97–1.01)	0.99 (0.97–1.01)
Parity				
Nulliparous	141 538	10 291	1.00	1.00
Parous	1 168 265	78 838	0.94 (0.92–0.96)	0.92 (0.90–0.94)
No. of children‡				
1	185 571	13 178	1.00	1.00
2	550 543	33 614	0.92 (0.90–0.94)	0.96 (0.94–0.98)
3	282 728	19 341	0.95 (0.93–0.97)	0.95 (0.93–0.97)
4+	149 423	12 705	1.06 (1.03–1.08)	0.98 (0.95–1.00)
Duration of oral contraceptive use				
Never	527 767	45 956	1.00	1.00
<5 yrs	308 821	17 207	1.01 (0.99–1.02)	1.03 (1.01–1.05)
5+ yrs	428 712	22 350	0.98 (0.96–0.99)	1.00 (0.99–1.02)
HT use				
Never	643 484	46 799	1.00	1.00
Ever	652 768	41 053	1.04 (1.02–1.05)	1.07 (1.06–1.09)

CI = confidence interval; HT = hormone therapy; RR = relative risk.

∗ Estimates of RR are adjusted by age and region of residence.

† Estimates of RR are adjusted by age and region of residence plus deprivation, educational attainment, smoking, BMI, alcohol, strenuous physical activity, treatment for diabetes, age at menarche, parity, duration of oral contraceptive use, and HT use as appropriate.

‡ In 1 168 265 women who had had at least 1 child.
